# Retraction Note: Restored microRNA-133a-3p or Depleted PSAT1 Restrains Endothelial Cell Damage-Induced Intracranial Aneurysm Via Suppressing the GSK3β/β-Catenin Pathway

**DOI:** 10.1186/s11671-023-03833-5

**Published:** 2023-03-20

**Authors:** Qiang Jia, Shixin Yan, Jie Huang, Shixin Xu

**Affiliations:** 1 Department of Neurosurgery, Tianjin Huanhu Hospital, Tianjin, 300050 Tianjin China; 2 Department of Radiology, Tianjin Huanhu Hospital, Tianjin, 300050 Tianjin China; 3 Department of Neurology, Cangzhou People’s Hospital, 20 North Street, Cangzhou, 061000 Hebei China; 4 Clinical Laboratory, First Teaching Hospital of Tianjin University of Traditional Chinese Medicine, 314 An shan xin Road, Nan Kai District, Tianjin, 300000 Tianjin China

**Retraction note: Nanoscale Research Letters (2020) 15:177** **https://doi.org/10.1186/s11671-020-03396-9**

The Editors in Chief have retracted this article. After publication, concerns were raised about the authors' contributions and integrity of data. The authors did not respond to the request to provide raw data and the original ethics approval. The Editors in Chief, therefore, have lost confidence in the findings of this article. The authors did not respond to any correspondence from the Editors about this retraction.

